# Showing Love to Letters: Improving the Completeness and Clarity of Care Plan in Patient Discharge Letters

**DOI:** 10.1192/bjo.2026.11361

**Published:** 2026-06-30

**Authors:** Eberechi Chris-Okoro

**Affiliations:** Hertfordshire Partnership NHS Foundation Trust, Hatfield, United Kingdom. QIClearn, London, United Kingdom

## Abstract

**Aims:**

Sketchy discharge summaries are common, impacting on patients' ability to maintain a shared understanding of factors leading to their deterioration and their recovery. This project was designed to improve the quality of patient discharge summary letters from a psychiatry ward to achieve eight out of ten letters having all relevant categories of important information included and a clear support plan.

**Methods:**

The project was conducted over a three-month period. Firstly, baseline diagnostics of the cause and extent of the problem was completed through observation, discussion with patients and colleagues, as well as use of tools like process map, fishbone diagram, and bar chart. The Model for Improvement informed design of the project based on these diagnostics. An aim was set, and a robust definition of measures was created with inclusion and exclusioncriteria. I completed a baseline run chart, sampling 20 discharge summaries. This gave me a median baseline to establish any statistically significant changes following implemented changes and subsequent sampling of all (20) further discharge summaries completed throughout the project. Using the Plan–Do–Study–Act (PDSA) methodology, I captured change ideas with my team and multi-faceted interventions were implemented. The discharge template was adjusted to include prompts for all relevant categories of the discharge summary. Additional interventions included early allocation of doctors to discharge summaries and education (via email, team doctors’ meeting, induction document about writing discharge letters, one-to-one teaching and feedback) for doctors writing discharge summaries.

**Results:**

The run chart showed that the targeted change ideas led to a statistically significant shift in the data (more than six data points above the median). Nine out of ten summaries were complete and had a detailed support plan. There was a sustained pattern of change above the median baseline.

**Conclusion:**

This quality improvement project resulted in significantly increased proportion of complete discharge summary letters, illustrating the merit of utilizing quality improvement methodology alongside team engagement to improve clinical outcomes. Continued oversight will be needed to ensure changes are sustained. This project is being shared with other wards, aiming for wider implementation of changes.

